# The elevated lactate dehydrogenase to albumin ratio is a risk factor for developing sepsis-associated acute kidney injury: a single-center retrospective study

**DOI:** 10.1186/s12882-024-03636-5

**Published:** 2024-06-19

**Authors:** Yipeng Fang, Yuan Zhang, Xin Zhang

**Affiliations:** 1 Laboratory of Molecular Cardiology, The First Affiliated Hospital of Shantou University Medical College, 57th Changping Road, Shantou, Guangdong Province 515041 People’s Republic of China; 2 Laboratory of Medical Molecular Imaging, The First Affiliated Hospital of Shantou University Medical College, Shantou, Guangdong Province People’s Republic of China; 3 Shantou University Medical College, Shantou, Guangdong Province People’s Republic of China

**Keywords:** Lactate dehydrogenase, Serum albumin, Acute renal failure, Acute renal insufficiency, Novel biomarker, Sepsis-3, Risk factors

## Abstract

**Background:**

There is no evidence to determine the association between the lactate dehydrogenase to albumin ratio (LAR) and the development of sepsis-associated acute kidney injury (SAKI). We aimed to investigate the predictive impact of LAR for SAKI in patients with sepsis.

**Methods:**

A total of 4,087 patients with sepsis from the Medical Information Mart for Intensive Care IV (MIMIC IV) database were included. Logistic regression analysis was used to identify the association between LAR and the risk of developing SAKI, and the relationship was visualized using restricted cubic spline (RCS). The clinical predictive value of LAR was evaluated by ROC curve analysis. Subgroup analysis was used to search for interactive factors.

**Results:**

The LAR level was markedly increased in the SAKI group (*p* < 0.001). There was a positive linear association between LAR and the risk of developing SAKI (p for nonlinearity = 0.867). Logistic regression analysis showed an independent predictive value of LAR for developing SAKI. The LAR had moderate clinical value, with an AUC of 0.644. Chronic kidney disease (CKD) was identified as an independent interactive factor. The predictive value of LAR for the development of SAKI disappeared in those with a history of CKD but remained in those without CKD.

**Conclusions:**

Elevated LAR 12 h before and after the diagnosis of sepsis is an independent risk factor for the development of SAKI in patients with sepsis. Chronic comorbidities, especially the history of CKD, should be taken into account when using LAR to predict the development of AKI in patients with sepsis.

**Supplementary Information:**

The online version contains supplementary material available at 10.1186/s12882-024-03636-5.

## Background

Sepsis, defined by the Sepsis 3.0 definition as organ dysfunction caused by an imbalanced host response to infection, is common in intensive care units (ICUs) [1]. Acute kidney injury (AKI) is one of the most common complications of sepsis, and its prevalence increases progressively with the aggravation of sepsis [2]. However, epidemiological studies investigating the prevalence of sepsis-associated AKI (SAKI) are still limited. The incidence of SAKI is approximately 10 million (1.4 per 1000 population) per year [3]. Due to the low detection rate of SAKI, the actual percentage in real life would be even higher [3]. The high mortality rate of SAKI is also a major public health concern. Compared to patients with AKI induced by other causes, patients with SAKI had a higher risk of in-hospital mortality and longer hospital stays [4]. Although SAKI has received much attention and remarkable progress has been made in SAKI treatment, the mortality of SAKI remains unacceptably high [5]. As early reversal of SAKI is associated with decreased morality [6], early identification of sepsis patients at high risk of SAKI is crucial.

Lactic dehydrogenase (LDH) is one of the key enzymes involved in glycolysis and is widely distributed in various tissues and organs throughout the human body [7]. LDH acts as a catalyst for the conversion of pyruvate to lactic acid. Under hypoxic conditions, activation of LDH catalyzes the conversion of pyruvate to lactate during glycolysis [8]. When cell damage occurs, LDH could be released into the bloodstream from LDH-containing cells, leading to an increase in serum LDH levels. It has been shown to be an independent risk factor for poor outcome in sepsis [9], acute pancreatitis [10], acute kidney disease [11] and tumors [12]. However, evidence regarding the predictive value of LDH for AKI is still limited. Two studies found that elevated LDH was an independent risk factor for AKI after cardiac surgery [13, 14]. According to the study by Amitai I et al., LDH > 380 U/L and albumin < 3.6 gr/dLwere significantly associated with the development of high-dose methotrexate-induced AKI [15]. However, the predictive role of LDH in the development of SAKI remains unclear.

Serum albumin is a commonly used laboratory parameter for assessing patients’ nutritional status and renal injury, especially in chronic disease [16, 17]. Podocyte injury can disrupt the integrity of the glomerular filtration barrier, leading to proteinuria and hypoproteinemia [18]. Previous evidence suggests that decreased serum albumin is associated with higher mortality in sepsis and AKI cohorts [19, 20]. In addition, serum albumin can serve as a predictor of AKI [21]. Several combined indicators with albumin showed strong predictive and prognostic value for AKI, such as the fibrinogen to albumin ratio [22], uric acid to albumin ratio [23] and lactate to albumin ratio [24].

Overall, serum LDH and albumin have shown potential as biomarkers for the diagnosis and prognosis of AKI. A recent study has reported that the combination of LDH and albumin showed a better prognostic value in patients with AKI than the use of either parameter alone [25]. However, the predictive value of LDH combined with albumin for SAKI has not been investigated. In the present study, we aimed to investigat the relationship between the lactate dehydrogenase to albumin ratio (LAR) and the development of SAKI, as well as the predictive value of LAR for SAKI.

## Methods and materials

### Study design

This is a descriptive, retrospective, single-center study using hospital information from the Medical Information Mart for Intensive Care IV (MIMIC-IV database, Vision 2.1). The MIMIC-IV database contains information on approximately 180,000 patients, including 50,934 with ICU admission records, between 2008 and 2019 at the Beth Israel Deaconess Medical Center in Boston, Massachusetts, USA. It is a public database developed by the MIT Lab for Computational Physiology. Author FYP (certification number 43,025,968) was responsible for the initial data extraction using PgAdmin4 and PostgreSQL (version 9.6) software. Present manuscript was reported in accordance with the STROBE guidelines [26].

### Population

Adult patients with sepsis during their ICU stay were initially screened according to the Sepsis 3.0 criteria. The following patients were excluded from the initial cohort: (1) patients with recurring hospital admissions (only the first ICU admission record was included); (2) patients with the development of AKI before sepsis diagnosis; (3) patients who died within 48 h of ICU admission or had a length of ICU stay less than 48 h; and (4) patients with missing LDH or albumin results 12 h before or after sepsis diagnosis.

### Variable

The present study included demographic data, comorbidities, vital signs, laboratory parameters, disease severity scores and microbiological culture results. Demographics included age, sex, ethnicity and body weight. Comorbidities included hypertension, coronary heart disease, congestive heart failure, diabetes mellitus, chronic lung disease, liver disease, chronic kidney disease (CKD) and malignant cancer. Vital signs, including heart rate, respiratory rate, mean arterial pressure (MAP), temperature and oxygen saturation (SpO_2_), were measured at the time of sepsis diagnosis. The initial values of laboratory parameters obtained after sepsis diagnosis were used in the final analysis, including serum white blood cell (WBC) count, hemoglobin, platelets, creatinine (SCr), blood urea nitrogen (BUN), sodium, potassium, chloride and lactate levels. The initial Simplified Acute Physiology Score II (SAPS II) and Glasgow Coma Scale (GCS) at the time of ICU admission were used, while the initial value of the Sequential Organ Failure Assessment (SOFA) score at the time of sepsis diagnosis was used. Since LDH is strongly associated with lactate formation and liver dysfunction, the level of lactic acid and liver injury-related markers at the time of sepsis diagnosis, including bilirubin, alanine transaminase (ALT) and aspartate transaminase (AST), were also included in the final analysis.

### Exposure and clinical outcomes

The exposure was the value of LAR (IU/g). Only the results of LDH (IU/L) and albumin (g/L) obtained 12 h before or after the diagnosis of sepsis were retained. If multiple values were available, the mean value was used to calculate the LAR value (IU/g). Patients were divided into four equal subgroups according to their LAR values: Q1, LAR ≤ 6.5 IU/g; Q2, 6.5 IU/g < LAR ≤ 9.3 IU/g; Q3, 9.3 IU/g < LAR ≤ 15.2 IU/g; and Q4, LAR ≥ 15.2 IU/g.

The development of AKI after sepsis was the primary outcome. AKI was diagnosed and stratified only on the basis of the change in plasma creatinine according to the KIDGO guidelines as reported in previous studies [27, 28]. SAKI was defined as the presence of AKI within the first 48 h after sepsis diagnosis. The secondary outcomes included RRT after sepsis diagnosis, the use of vasoactive drugs within 48 h of sepsis diagnosis, mortality indicators and LOS indicators.

### Data cleaning

Scatter plots were drawn to check for outliers. Outliers were handled as missing values. As the percentage of missing values was less than 10%, median/mean imputation was used for most variables. Missing lactic acid values, constituting 13% of the data, were imputed using a regression method based on the specified regression equation. Indicators with missing data exceeding 15% were omitted from the analysis. Details concerning the missing values are provided in Table S1.

$$Predicted lactic acid=0.838+0.006\times age+0.193\times SOFA$$

$$+0.147\times liver disease+0.005\times MBP+0.413\times$$

$$vasopressor-0.483\times CKD-0.014\times bilirubin$$

### Statistical analysis

Normally distributed variables were shown as the mean ± standard deviation (SD), and nonnormal variables were shown as the median and interquartile range (IQR). Categorical variables were presented as numbers and percentages. For pairwise comparisons of continuous variables, Student’s t test (for normally distributed values) and the Mann–Whitney U test (for nonnormally distributed values) were performed. For multiple comparisons of continuous variables, one-way ANOVA (for normally distributed values) and the Kruskal‒Wallis H tests (for nonnormally distributed values) were used. For categorical variables, the chi-square test was used. Univariate and multivariate logistic regression models were developed to investigate the predictive value of the LAR value for SAKI development. We evaluated multicollinearity among candidate variables using variance inflation factors (VIF), considering a VIF greater than 10 as indicative of significant collinearity [29]. To address significant multicollinearity, variables with a VIF exceeding 10 were converted into categorical variables based on their median or mean values. The VIF details for all variables are presented in Table S2. The receiver operating characteristic (ROC) curve and area under the ROC curve (AUC) were examined to determine the clinical predictive value of LAR for SAKI development. Restricted cubic spline (RCS) was performed to determine the linear and nonlinear relationship between the LAR value and the risk of developing SAKI. Subgroup analysis was performed to verify the robustness of the initial results and to find potential interactive factors. We performed all statistical analyses using Stata (version 15.1) and R software (version 4.1.3). A two-sided p value < 0.05 was considered statistically significant.

## Results

### Population and baseline information

**Fig. 1:**
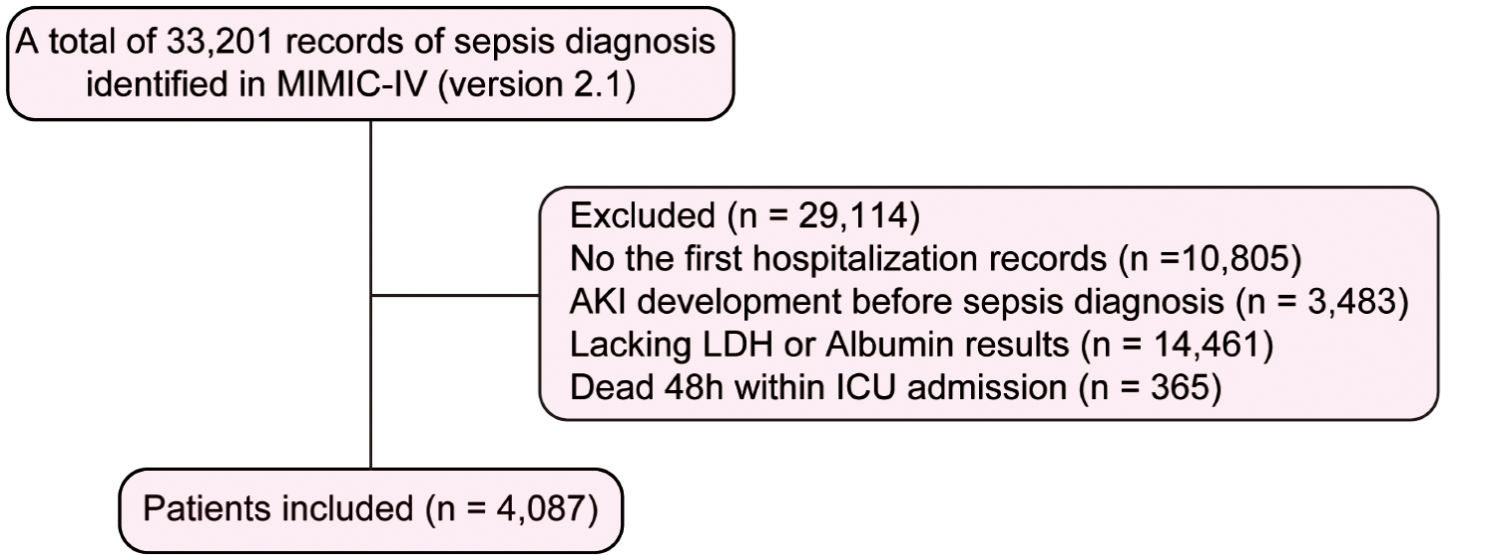
Flow chart. Flow chart of patient selection

In the MIMIC-IV database (Vision 2.1), there were 22,396 adult patients meeting the Sepsis-3.0 criterion during their ICU stay. A total of 4,087 patients were included in our final analysis. The flow chart of population screening is shown in Fig. 1. The incidence of SAKI was 26.25% (1,073/4,087). As shown in Table 1, there was no significant difference in age between the SAKI and non-SAKI subgroups (*p* = 0.455). Patients with SAKI were more likely to be men but less likely to be Caucasians. Body weight was higher in the SAKI subgroup. Patients with SAKI had a higher proportion of comorbid coronary heart disease, congestive heart failure, diabetes mellitus, liver disease and CKD but a lower proportion of comorbid hypertension. Patients in the SAKI subgroup had higher values for heart rate, respiratory rate, SOFA score, SAPS II score, WBC and potassium but lower values for MAP, temperature, GCS score, platelets, sodium and chloride. Patients in SAKI subgroup also had higher values for lactic acid, bilirubin, ALT and AST than those in non-SAKI subgroup (all *p* < 0.001). They were also more likely to have a positive culture and using vasopressor during their ICU stay. Levels of LDH (348 (235, 618) vs. 265 (199, 392)) and LAR (12.5 (7.7, 22.4) vs. 8.6 (6.2, 13.5)) were higher in the SAKI subgroup, whereas albumin (30.0 (25.0, 34.0) vs. 31.0 (27.0, 36.0)) was decreased in the SAKI subgroup (all *p* < 0.05).

**Table 1:** The baseline information and clinical characteristic of patients with and without SAKI

Variable	Total	Non-SAKI	SAKI	*p*-value
Number	4,087	3,014	1,073	NA
Age (years)	64.3 ± 17.3	64.2 ± 17.6	64.7 ± 16.2	0.455
Male (%)	2,285 (55.84)	1,642 (54.48)	640 (59.65)	0.003
Ethnicity, white (%)	2,444 (59.80)	1,839 (61.02)	605 (56.38)	0.008
Weight (kg)	82.2 ± 25.4	80.7 ± 24.9	86.2 ± 26.1	< 0.001
Comorbidities				
Hypertension (%)	1,481(36.24)	1,148(38.09)	333(31.03)	< 0.001
Coronary heart disease (%)	692(16.93)	460(15.26)	232(21.62)	< 0.001
Congestive heart failure (%)	1,119(27.38)	730(24.22)	389(36.25)	< 0.001
Diabetes mellitus (%)	1,207(29.53)	830(27.54)	377(35.14)	< 0.001
Chronic pulmonary disease (%)	1,029(25.18)	747(24.78)	282(26.28)	0.332
Liver disease (%)	1,023(25.03)	679(22.53)	344(32.06)	< 0.001
Chronic kidney disease (%)	896(21.92)	529(17.55)	367(34.20)	< 0.001
Malignant cancer (%)	629(15.39)	480(15.93)	149(15.93)	0.112
Vital sign				
Heart rate (bpm)	90.2 ± 20.5	89.3 ± 19.9	92.5 ± 22.1	< 0.001
Respiratory rate (bpm)	20.3 ± 5.9	20.1 ± 5.7	21.1 ± 6.4	< 0.001
MAP (mmHg)	77.3 ± 15.9	77.7 ± 15.6	76.3 ± 16.6	0.013
SpO2 (%)	97(95,100)	97(95,99)	98(95,100)	0.466
Temperature (℃)	36.9 ± 0.9	36.9 ± 0.8	36.7 ± 1.1	< 0.001
Disease severity score, point				
SOFA score	7(4,10)	6(4,8)	10(7,13)	< 0.001
SAPS II	39(30,48)	36(29,45)	47(38,58)	< 0.001
GCS	14(9,15)	14(10,15)	12(6,14)	< 0.001
Laboratory parameters				
WBC (k/uL)	11.0(7.4,15.7)	10.6(7.2,15.1)	11.7(7.9,17.44)	< 0.001
Hemoglobin (g/dL)	10.3 ± 2.1	10.3 ± 2.0	10.2 ± 2.2	0.107
Platelets (k/uL)	162(104,233)	166(106,236)	152(100,220)	0.002
Sodium (mmol/L)	138.3 ± 6.0	138.6 ± 5.8	137.3 ± 6.4	< 0.001
Potassium (mmol/L)	4.1 ± 0.7	4.0 ± 0.7	4.4 ± 0.9	< 0.001
Chloride (mmol/L)	104.7 ± 7.0	105.3 ± 6.7	103.0 ± 7.6	< 0.001
Liver function parameters				
Lactic acid (mmol/L)	2.4(1.6,3.5)	2.2(1.6,3.1)	2.9(1.8,5.3)	< 0.001
Bilirubin (mg/dL)	0.8(0.5,2.1)	0.8(0.5,1.9)	1.0(0.5,2.6)	< 0.001
ALT (IU/L)	33(19,80)	32(18,71)	40(21,123)	< 0.001
AST (IU/L)	51(28,129)	47(26,107)	72(33,216)	< 0.001
Creatinine (mg/dL)				
Baseline 48 h	1.0(0.7,1.6)	0.9(0.7,1.4)	1.5(0.9,2.7)	< 0.001
Baseline 7 day	1.0(0.7,1.6)	0.9(0.7,1.4)	1.4(0.9,2.7)	< 0.001
Within 48 h of sepsis diagnosis	1.1(0.7,1.8)	0.9(0.7,1.3)	2.0(1.3,3.5)	< 0.001
LAR and related parameters				
LDH (IU/L)	282(206,438)	265(199,392)	348(235,618)	< 0.001
Albumin (g/L)	31.0(26.0,35.3)	31.0(27.0,36.0)	30.0(25.0,34.0)	< 0.001
LAR (IU/g)	9.3(6.5,15.2)	8.6(6.2,13.5)	12.5(7.7,22.4)	< 0.001
Culture positive	2,145(52.48)	1,491(49.47)	654(60.95)	< 0.001
Sputum	809(19.79)	491(16.29)	318(29.64)	< 0.001
Urine	962(23.54)	671(22.26)	291(27.12)	0.001
Blood	576(14.09)	403(13.37)	173(16.12)	0.026
Vasopressor use (%)	1,396(34.16)	829(27.50)	567(52.84)	< 0.001

a. Continuous variables are displayed as mean (standard deviation) or median (first quartile–third quartile); categorical variables are displayed as count (percentage); ALT, alanine transaminase; AST, aspartate transaminase; GCS, Glasgow Coma Scale; LAR, lactate dehydrogenase to serum albumin ratio; LDH, lactate dehydrogenase; MAP, mean arterial pressure; SAPS, Simplified Acute Physiology Score; SOFA, Sequential Organ Failure Assessment; SpO2, oxygen saturation; WBC, white blood cell

### Linear relationship between log_2_ (LAR) value and SAKI development

**Fig. 2:**
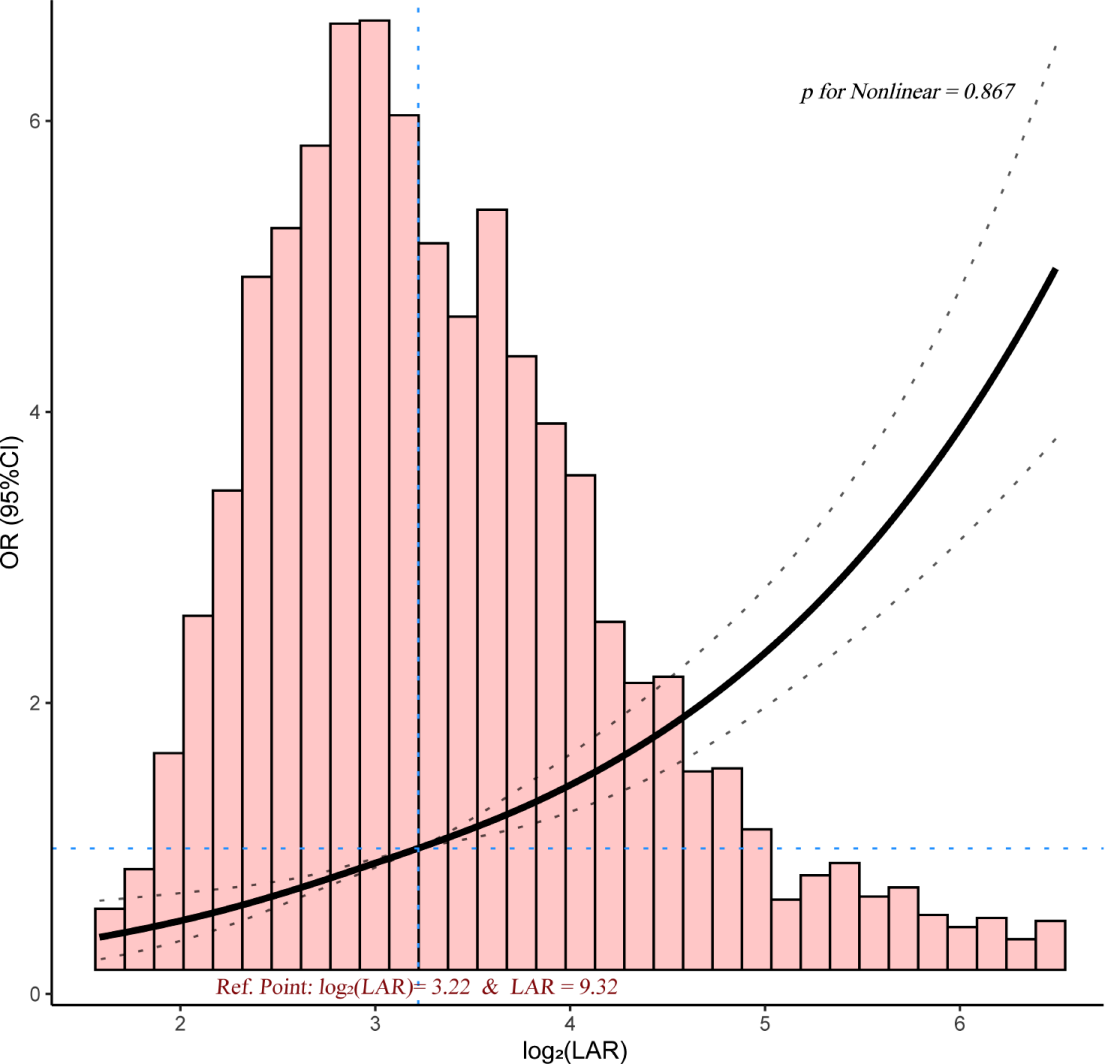
Restricted cubic spline. There was a positive linear relationship between log_2_ (LAR) and the risk of developing SAKI in patients with sepsis

Figure 2 shows the relationship between the log_2_ (LAR) value and SAKI development in patients with sepsis in the ICU using the RCS technique. A positive linear relationship was detected (p for nonlinearity = 0.867), with the reference point log_2_ (LAR) = 3.22 (LAR = 9.32). An LAR value higher than 9.32 IU/g was considered a risk factor for the development of SAKI (OR > 1). Therefore, an LAR of 9.32 IU/g was used as the cutoff point to generate the high-LAR and low-LAR subgroups for the subgroup analysis.

### LAR categories and clinical outcomes

Patients were further divided into four categories according to the IQR of the LAR value (Q1 –Q4 categories). The crude outcomes of those four subgroups are shown in Table 2. The incidence rate of SAKI increased with increasing LAR, with the incidence rate increasing stepwise from Q1 (15.82%) to Q4 (41.35%, *p* < 0.001). In SAKI patients, the incidence rate of stage 2 and 3 SAKI also increased stepwise from Q1 (24.22%) to Q4 (50.12%, *p* < 0.001). In the SAKI subgroups, more RRT was performed in the Q4 category (LAR > 15.2 IU/g), but no difference was observed from Q1 to Q4. No significant difference was found in the time interval from sepsis to developing SAKI and from SAKI to using RRT among Q1 –Q4 categories (*p* = 0.854 and 0.242). Increasing trends were also observed for vasopressor use, 28-day mortality, 90-day mortality, hospital mortality, ICU mortality, hospital-LOS and ICU-LOS (all *p* < 0.001). Patients in the Q4 category had the highest 28-day mortality rate (33.33%), followed by Q3 (22.24%), Q2 (15.18%) and Q1 (8.64%).

**Table 2:** Comparisons of the clinical outcomes among patients in different LAR categories

Outcomes	Q1(LAR ≤ 6.5)	Q2(6.5 < LAR ≤ 9.3)	Q3(9.3 < LAR ≤ 15.2)	Q4(LAR ≥ 15.2)	*p*-value
Number	1,018	1,021	1,025	1,023	NA
SAKI (%)	161(15.82)	228(22.33)	261(25.46)	423(41.35)	< 0.001
SAKI Stage (%)					< 0.001
Stage 1	122(75.78)	165(72.37)	162(62.07)	211(49.88)	
Stage 2 + 3	39(24.22)	63(27.63)	99(37.63)	212(50.12)	
RRT (%)	42(26.09)	57(25.00)	66(25.29)	169(39.95)	< 0.001
Time interval					
From sepsis to developing SAKI (h)	23(11,35)	24(13,35)	23(10,36)	24(12,35)	0.854
From SAKI to using RRT (h)	15(3,37)	14(3,51)	21(5,72)	22(4,68)	0.242
Vasoactive drugs use (%)	217(21.32)	313 (30.66)	401 (39.12)	465 (45.45)	< 0.001
28-day mortality (%)	88(8.64)	155(15.18)	228(22.24)	341(33.33)	< 0.001
90-day mortality (%)	147(14.44)	242(23.70)	305(29.76)	417(40.76)	< 0.001
Hospital mortality (%)	49(4.81)	98(9.60)	150(14.63)	258(25.22)	< 0.001
ICU mortality (%)	32(3.14)	61(5.97)	111(10.83)	207(20.23)	< 0.001
Hospital LOS (%)	6.7(4.4,11.6)	7.9(5.0,13.0)	9.1(5.8,16.1)	11.3(6.2,19.5)	< 0.001
ICU LOS (%)	2.3(1.4,4.2)	2.9(1.6,5.4)	3.6(1.9,7.0)	4.6(2.5,9.6)	< 0.001

a. Continuous variables are displayed as mean (standard deviation) or median (first quartile–third quartile); categorical variables are displayed as count (percentage); RRT, renal replacement therapy; ICU, intensive care unit; LAR, lactate dehydrogenase to serum albumin ratio; LOS, length of stays; SAKI, sepsis-associated acute kidney injury

Logistic regression models were performed to further determine the crude and adjusted effects of LAR on the development of SAKI. Table 3 shows that in one original model and three adjusted models, each IU/g increase in LAR was significantly associated with a 1–2% increase in the risk of SAKI development (OR: 1.001–1.002, *p* < 0.001). The risk of SAKI increased stepwise from Q2 (OR 1.53, 95% CI 1.22–1.91, *p* < 0.001) to Q4 (OR 3.75, 95% CI 3.04–4.63, *p* < 0.001) in the unadjusted model, using the Q1 category as a reference. After adjustment for potential confounders, a similar trend was observed, but the Q2 and Q3 categories lost statistical significance in Model 3 (*p* = 0.259 and 0.152). Elevated LAR was also considered an independent risk factor for RRT in patients with SAKI (all OR > 1, *p* < 0.001). When LAR was divided into four categories, only extremely high LAR, the Q4 category (LAR > 15.2 IU/g), was identified as an independent risk factor for RRT, taking the Q1 category (LAR ≤ 6.5 IU/g) as a reference. This significant effect was lost after adjusting potential confounders in Model 3 (*p* = 0.065). In addition, elevated LAR was identified as an independent risk factor for 28-day and in-hospital mortality in all models (all OR > 1, *p* < 0.01). The risk of mortality tended to increase steadily with increasing LAR level from Q2 (OR 1.42, 95% CI 1.05–1.90, *p* = 0.021 for 28-day mortality; OR 0.157, 95% CI 1.05–2.29, *p* = 0.017 for in-hospital mortality) to Q4 (OR 3.32, 95% CI 2.49–4.42, *p* < 0.001 for 28-day mortality; OR 3.88, 95% CI 2.73–5.52, *p* < 0.001 for in-hospital mortality) in model 3, with the Q1 category (LAR ≤ 6.5 IU/g) as a reference.

**Table 3:** Logistic regression model for clinical outcomes of patients in different LAR categories

	Unadjusted		Model 1		Model 2		Model 3	
Variable	OR (95% CI)	*p*-value	OR (95% CI)	*p*-value	OR (95% CI)	*p*-value	OR (95% CI)	*p*-value
SAKI								
LAR	1.02(1.01–1.02)	< 0.001	1.02(1.01–1.02)	< 0.001	1.01(1.01–1.02)	< 0.001	1.01(1.01–1.02)	< 0.001
Q1 (LAR ≤ 6.5)	1(Ref)		1(Ref)		1(Ref)		1(Ref)	
Q2 (6.5 < LAR ≤ 9.3)	1.53 (1.22–1.91)	< 0.001	1.53(1.22–1.92)	< 0.001	1.37(1.09–1.73)	0.008	1.15(0.90–1.47)	0.259
Q3 (9.3 < LAR ≤ 15.2)	1.82(1.46–2.26)	< 0.001	1.84(1.48–2.30)	< 0.001	1.67(1.33–2.10)	< 0.001	1.19(0.94–1.52)	0.152
Q4 (LAR > 15.2)	3.75(3.04–4.63)	< 0.001	3.87(3.13–4.77)	< 0.001	3.43(2.74–4.30)	< 0.001	1.92(1.50–2.45)	< 0.001
RRT in SAKI subgroup								
LAR	1.01(1.00-1.01)	< 0.001	1.01(1.00-1.01)	< 0.001	1.01(1.00-1.01)	< 0.001	1.01(1.00-1.01)	< 0.001
Q1 (LAR ≤ 6.5)	1(Ref)		1(Ref)		1(Ref)		1(Ref)	
Q2 (6.5 < LAR ≤ 9.3)	0.94(0.59–1.50)	0.808	0.99(0.62–1.58)	0.960	0.99(0.61–1.62)	0.968	0.86(0.52–1.44)	0.569
Q3 (9.3 < LAR ≤ 15.2)	0.96(0.61–1.50)	0.855	1.02(0.65–1.60)	0.947	1.37(0.84–2.22)	0.209	0.99(0.60–1.66)	0.981
Q4 (LAR > 15.2)	1.89(1.26–2.82)	0.002	1.87(1.24–2.80)	0.003	2.61(1.66–4.08)	< 0.001	1.59(0.97–2.59)	0.065
28-day mortality								
LAR	1.01(1.00-1.01)	< 0.001	1.01(1.00-1.01)	< 0.001	1.00(1.00-1.01)	< 0.001	1.01(1.01–1.01)	< 0.001
Q1 (LAR ≤ 6.5)	1(Ref)		1(Ref)		1(Ref)		1(Ref)	
Q2 (6.5 < LAR ≤ 9.3)	1.89(1.43–2.50)	< 0.001	1.80(1.36–2.38)	< 0.001	1.64(1.23–2.17)	0.001	1.42(1.05–1.90)	0.021
Q3 (9.3 < LAR ≤ 15.2)	3.02(2.32–3.93)	< 0.001	3.05(2.34–3.98)	< 0.001	2.67(2.04–3.50)	< 0.001	2.07(1.56–2.75)	< 0.001
Q4 (LAR > 15.2)	5.28(4.10–6.81)	< 0.001	5.70(4.41–7.38)	< 0.001	4.57(3.50–5.96)	< 0.001	3.32(2.49–4.42)	< 0.001
Hospital mortality								
LAR	1.01(1.00-1.01)	< 0.001	1.01(1.00-1.01)	< 0.001	1.01(1.00-1.01)	< 0.001	1.01(1.00-1.01)	< 0.001
Q1 (LAR ≤ 6.5)	1(Ref)		1(Ref)		1(Ref)		1(Ref)	
Q2 (6.5 < LAR ≤ 9.3)	2.10(1.47–2.99)	< 0.001	2.03(1.43–2.90)	< 0.001	1.84(1.28–2.63)	0.001	1.57(1.08–2.29)	0.017
Q3 (9.3 < LAR ≤ 15.2)	3.39(2.42–4.74)	< 0.001	3.43(2.45–4.81)	< 0.001	2.97(2.11–4.18)	< 0.001	2.18(1.52–3.12)	< 0.001
Q4 (LAR > 15.2)	6.67(4.84–9.18)	< 0.001	7.10(5.14–9.81)	< 0.001	5.70(4.10–7.94)	< 0.001	3.88(2.73–5.52)	< 0.001

Model 1 = adjusting sex, age and ethnicity

Model 2 = Model 1 + adjusting white blood cells, platelet, hemoglobin, sodium and potassium, hypertension, coronary heart disease, heart failure, diabetes, liver disease, chronic pulmonary disease, chronic kidney disease, malignant cancer

Model 3 = Model 2 + adjusting lactic acid, bilirubin, ALT, AST, vasopressor use, mean blood pressure, GCS score, SOFA score and SAPSII score

CI: confidence interval; ALT, alanine transaminase; AST, aspartate transaminase; GCS, Glasgow Coma Scale; LAR; lactate dehydrogenase to albumin ratio; OR: odds ratio; SAKI, sepsis-associated acute kidney injury; SAPS, Simplified Acute Physiology Score; SOFA, Sequential Organ Failure Assessment

### Clinical predictive values

**Fig. 3:**
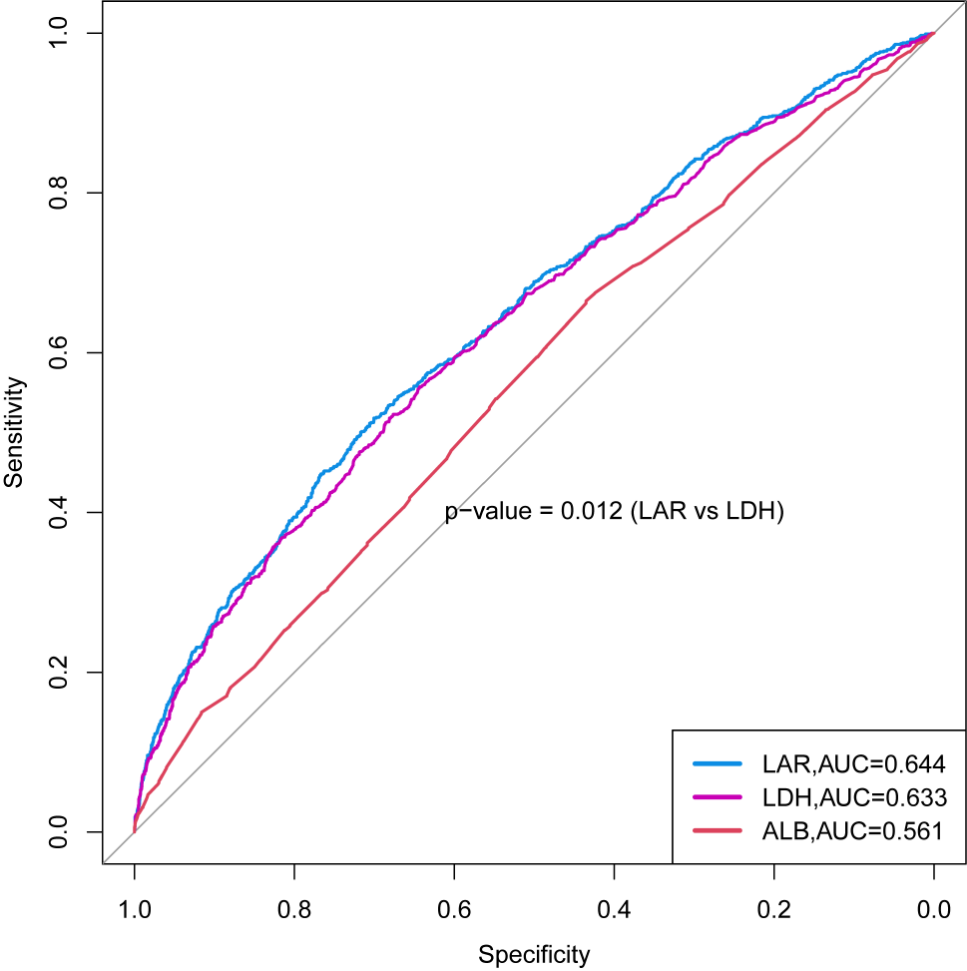
ROC curve. LAR (blue line) had a better predictive value for SAKI in patients with sepsis than LDH alone (purple line) and serum albumin alone (red line)

The clinical value of LAR in predicting SAKI was determined by ROC curve analysis. As shown in Fig. 3, LAR showed a moderate predictive ability for the development of SAKI (AUC 0.644, 95% CI 0.624–0.664). LAR had a significantly better predictive ability than LDH alone (AUC 0.633, 95% CI 0.614–0.653, *p* = 0.012). Albumin alone had the worst predictive ability (AUC 0.561, 95% CI 0.541–0.581).

### Subgroup analysis and post hoc analysis

**Fig. 4:**
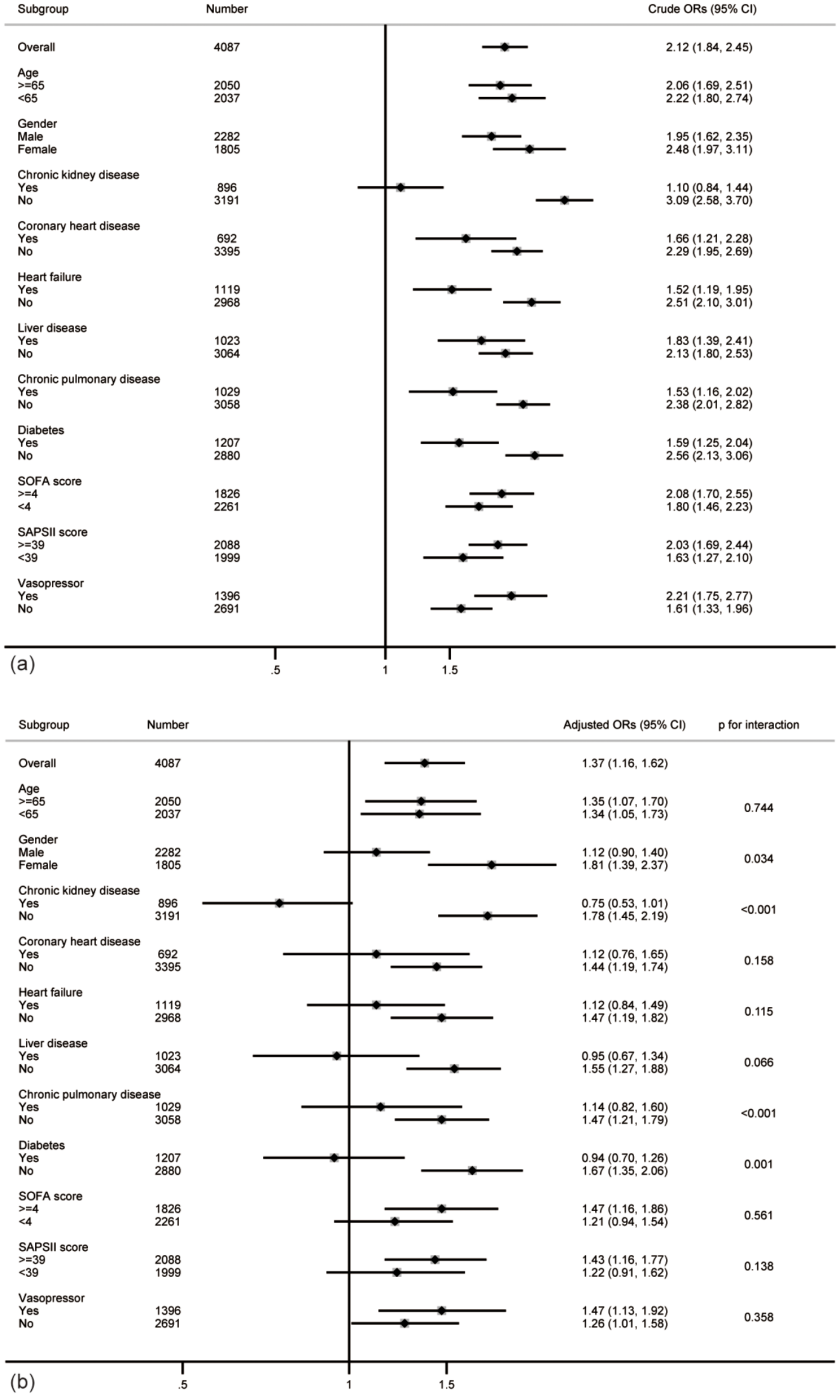
Subgroup analysis. The association between LAR and the risk of SAKI development was detected in the crude model (**a**) and adjusted model (**b**). Subgroup analysis indicated that there were significant ‘LAR×sex’, ‘LAR×CKD’, ‘LAR×pulmonary disease’ and ‘LAR×diabetes’ interactions

The relationship between the LAR value and SAKI development was still robust in different statuses (shown in Fig. 4). Four interactive factors were found, including sex, CKD, chronic pulmonary disease and diabetes (*p* for interaction < 0.05). It should be noted that the LAR value and SAKI showed a negative relationship in patients with a history of CKD, but no statistical significance was reached (OR 0.75, 95% CI 0.53–1.01, *p* = 0.064).

**Fig. 5:**
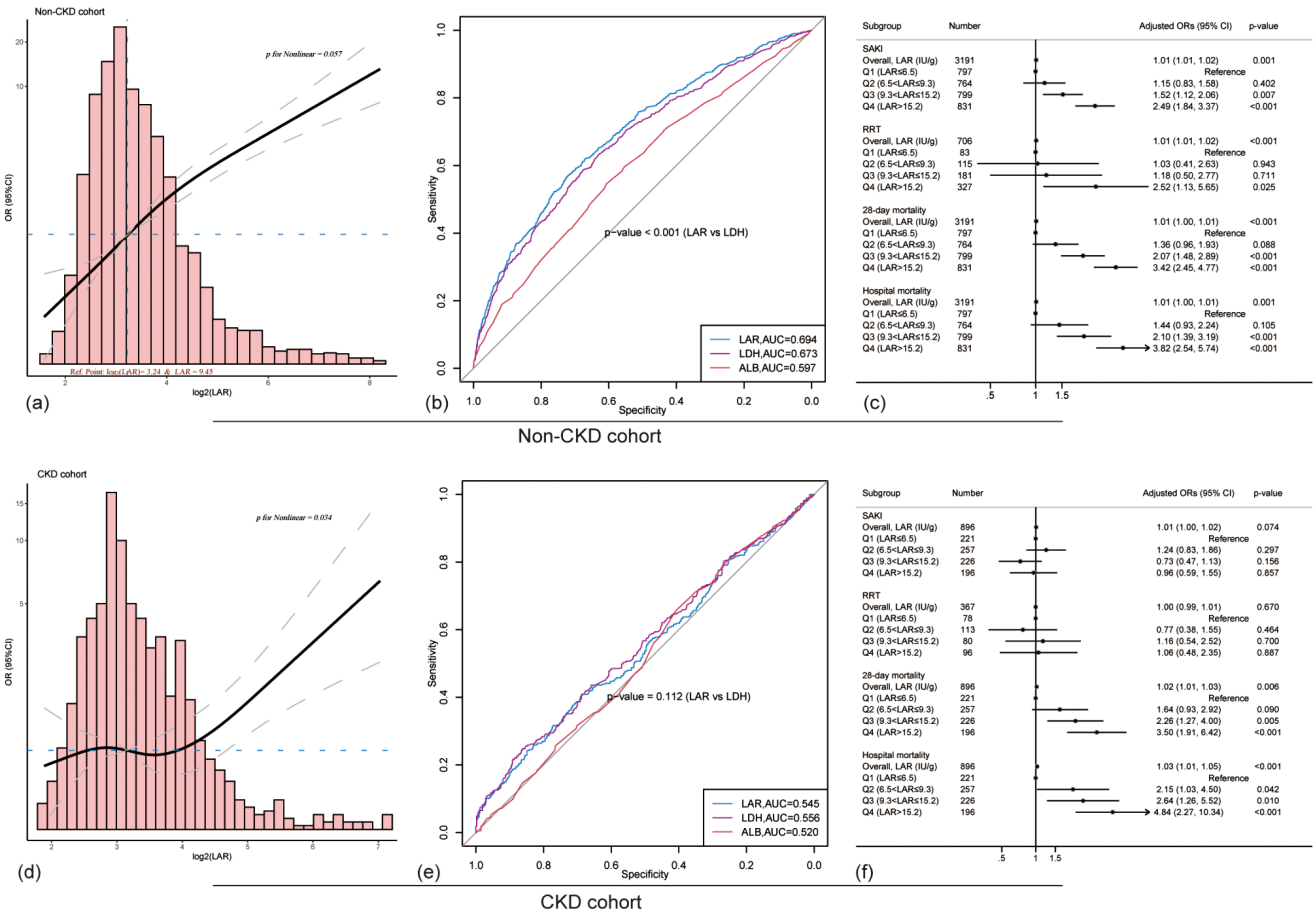
Post hoc analysis. The association between the LAR and the risk of SAKI development was further investigated in the non-CKD (**a**-**c**) and CKD cohorts (**d**-**f**) of patients with sepsis. (**d**) Restricted cubic spline showed that the positive linear relationship disappeared in sepsis patients with a history of CKD. (**e**) LAR had poor predictive value for SAKI development in sepsis patients with a history of CKD. (**f**) LAR was not associated with the risk of SAKI development in the adjusted logistic regression model

In the post hoc analysis, the relationship and predictive value between LAR and SAKI development in patients with or without a history of CKD were further determined. Figure 5a shows that a significant positive linear relationship between the LAR and SAKI development still existed in patients without CKD (*p* for nonlinear = 0.057). LAR showed moderate predictive value for SAKI (AUC 0.694), which was better than LDH alone (AUC 0.673) and albumin alone (AUC 0.597) (Fig. 5b). A similar trend was observed in logistic regression analysis in patients without a history of CKD compared with the overall cohort (Fig. 5c).

The above findings did not hold true for sepsis patients with a history of CKD. As shown in Fig. 5d, there was no significant trend between LAR and the development of SAKI in patients with a history of CKD (95% CI included 1). Although an extremely high LAR was associated with a higher risk of SAKI development, this was only confirmed by the very small sample. A poor clinical predictive value of LAR was detected in the ROC curve (AUC 0.545, Fig. 5e). In the adjusted logistic regression model, a high LAR showed no association with the development of SAKI and RRT, but the elevated LAR was independent risk factors for 28-day and in-hospital mortality (Fig. 5f).

## **Discussions**

In the present study, we found a positive linear correlation between LAR and the development of SAKI. Increased LAR was significantly associated with the risk of developing SAKI in patients with sepsis. LAR showed a moderate predictive value for the development of SAKI, which was superior to LDH or albumin alone. The LAR was also of significant value in identifying mortality. To interpret the value of LAR accurately, the history of CKD of the patients should not be ignored. The predictive values of LAR on predicting SAKI development did not exist in sepsis patients with a history of CKD but still existed in those without CKD. To the best of our knowledge, our study is the first to investigate the predictive value of LAR for the development of SAKI.

Serum albumin is a commonly used clinical indicator that reflects the nutritional status of patients and is involved in acute inflammatory responses [30]. Decreased serum albumin reflects poor nutrition and a potential systemic inflammatory reaction. Serum albumin plays several important roles in the development of AKI. First, albumin has been shown to have potent antioxidative stress and anti-inflammatory properties [30, 31]. Physiological level of albumin would help to alleviate renal tissue injury by downregulating oxidative stress and inhibiting proinflammatory reactions [32]. Second, as an important component of plasma colloid osmotic pressure, maintaining serum albumin in the physiological range would contribute to the regulation of fluid balance, intravascular volume and tissue perfusion [33], which may halt the process of AKI and loss of renal function. The product of albumin binding to platelet-activating factor and nitrogen oxide, named S-nitroso-albumin, helps maintain renal perfusion by dilating the local vascular network of the kidney [34]. Third, albumin may inhibit tissue damage by regulating several classical signaling pathways, like PI3K/AKT, NF-κB, MAPK and GSK-3β [35–38]. In renal proximal tubular cells (PTECs), activation of the PI3K/AKT pathway affects the level of albumin uptake by regulating PTEC protein reabsorption [35]. In the T2DM mouse model, recombinant albumin can effectively relieve endoplasmic reticulum stress and apoptosis by regulating the activation of the PI3K-AKT signaling pathway and alleviate T2DM by promoting glucose homeostasis and protecting islet β cells [36]. Pea albumin can inhibit DSS-induced colitis via inhibition of inflammation, regulation of NF-κB signaling and modulation of gut microbiota [37]. In addition, albumin inhibited serum starvation-induced mitochondrial damage, autophagy and apoptosis by regulating p38 MAPK and Akt/GSK-3β signaling [38]. Although there is no direct evidence to verify the potential signaling pathways involved in the effect of albumin on the development of AKI, the above classical signaling pathways have been widely demonstrated to be involved in the occurrence and development of AKI. In particular, albumin overload also leads to tissue injury [39]. Fourth, a normal serum albumin level is associated with a good physiological status of the body, reflecting the ability of humans to resist disease. Decreased serum albumin has been identified to be associated with several kinds of disease [39].

Many composite indicators using serum albumin are widely used in patients with sepsis and show higher predictive power than using parameters alone. The lactate-to-albumin ratio has a moderate predictive value for mortality in patients with sepsis or septic shock (AUC 0.74), with pooled sensitivity, specificity, and diagnostic odds ratios of 0.71, 0.68 and 5.23, respectively [40]. The blood urea nitrogen to albumin ratio (BAR) was found to be an independent risk factor for mortality in patients with sepsis (AUR 0.661) [41]. The red blood cell distribution width-to-albumin ratio (RAR) was reported as an independent risk indicator for 90-day mortality in elderly patients with AKI (AUC 0.656) [42]. Elevated RAR was independently associated with an increased risk of SAKI (RR 1.09) [43]. Combination with serum albumin is a commonly used method to improve the clinical value of indicators.

LDH is widely distributed enzyme across various cells and tissues. Elevated LDH levels indicate damage to the cell membrane and cell death, making it a common marker for predicting acute heart and liver injury in clinical settings [44, 45]. LDH is one of the key enzymes in glycolysis and is involved in lactate production. However, the diagnostic and prognostic value of LDH in patients with sepsis and SAKI has received much less attention. Its potential value as a predictive biomarker for nonsepsis-induced AKI has been demonstrated previously [13–15]. Elevated LDH was associated with multiple factors in the setting of AKI, as follows. First, as an important factor in the development of AKI, the activation of hypoxia-inducible factor (HIF) induced by hypoxia upregulates the transcription of LDH to promote glycolysis [46]. Second, LDH has been shown to be widely distributed in various tissues, including the kidney [13]. Renal tissue damage caused by inflammation, oxidative stress and ischemic/hypoxic events during AKI may lead to the release of intracellular LDH into the serum [47]. The above pathophysiological mechanisms are also relevant to the development of sepsis. In our present study, we found that patients with SAKI had higher LDH levels than those without SAKI. LDH alone was only moderately predictive of SAKI. As LDH is widely distributed and commonly elevated in many diseases, especially in acute myocardial injury, the value of using LDH alone to predict SAKI may be limited. Undeniably, the predictive value of LDH alone in AKI has been widely demonstrated in several AKI cohorts [13–15].

Recent studies have investigated the clinical value of LDH in combination with albumin. Lee BK et al. reported that an increased LAR was independently positively associated with hospital mortality in patients with lower respiratory tract infection, with good prognostic value (AUC = 0.808) [48]. In a nasopharyngeal carcinoma cohort, an LAR greater than 4.04 was associated with a 1.71-fold increased risk of death compared to those with a lower LAR [49]. Two recent studies have investigated the potential association between LAR and mortality in patients with sepsis. Deng YH et al. demonstrated a positive nonlinear relationship between LAR and mortality in critically ill patients with sepsis [25]. A similar trend of LAR on mortality was demonstrated in the AKI subgroup in patients with sepsis [50]. To date, the predictive value of LAR for SAKI remains unclear. Our results suggested a clear positive linear correlation between LAR and the risk of developing SAKI. LAR had a higher clinical prognostic value for SAKI than LDH alone and albumin alone in both crude and adjusted models.

To mitigate the effects of heart and liver tissue injuries on the predictive accuracy of LAR, we controlled for several variables including coronary heart disease, heart failure, liver disease, as well as levels of lactate, transaminase, and bilirubin in our multi-factor logistic regression analysis. Despite controlling for these potential confounders, LAR’s predictive significance for the development of SAKI remained substantial. However, caution is still warranted as our subgroup analysis revealed that an elevated LAR does not independently predict SAKI in patients suffering from liver disease or heart failure. This might be attributed to LDH being a non-specific indicator of tissue and cellular damage. Consequently, the diagnostic utility of LAR for SAKI could be compromised by increased LDH levels originating from cardiac and liver injuries. This highlights the necessity of considering the impact of these conditions when utilizing LAR as a predictor for SAKI.

Interestingly, a significant LAR-CKD interaction effect was observed. The prognostic value of LDH on SAKI was still robust in the non-CKD subgroup but disappeared in the CKD subgroup. We speculated that the possible mechanism might be due to the following aspects. Above all, chronic kidney injury may cause slow but constant damage to the cell and reduce the physiological reserve of LDH in the cell. We found that patients with CKD had lower levels of LDH (273 (208,410) vs. 284 (205,447), *p* = 0.064) and LAR (8.8 (6.5, 14.1) vs. 9.5 (6.5, 15.5), *p* = 0.024) than those without a history of CKD in the present cohort. In addition, proteinuria caused by podocyte injury significantly influences serum albumin levels and may also decrease the predictive value of LAR. More evidence is needed to support our speculation. Our findings also serve as a reminder that CKD history should not be ignored when using LAR to predict the development of AKI.

Our study possesses multiple strengths. Primarily, it utilizes data from a high-quality intensive care database with a substantial sample size, enhancing the accuracy and reliability of our findings. Additionally, the use of publicly accessible data mining codes from the MIMIC IV database (https://github.com/MIT-LCP/mimic-code) supports the precision and reproducibility of our results [51]. Our research rigorously evaluates the predictive capability of the leukocyte-to-albumin ratio (LAR) for acute kidney injury (AKI) in patients with sepsis. We carefully excluded patients who developed AKI prior to the diagnosis of sepsis to reduce potential confounding effects on the serum levels of LDH and albumin. We also considered several confounders, including acute cardiac injury, acute liver dysfunction, and chronic kidney disease (CKD). Notably, our post-hoc analysis revealed the influence of CKD on the clinical utility of LAR in forecasting sepsis-associated AKI (SAKI), which is critical for the effective application of LAR as a prognostic marker.

Admittedly, despite above benefits, some limitations do exist in present study, and the results should be interpreted with caution. First, this was a retrospective study using data from a single center, which may be subject to inherent bias. Second, selection bias seems inevitable because only patients with laboratory results of LDH and albumin were included in the final analysis. We think that patients with LDH and albumin results may be a different population from those without. Third, as dynamic change indicators, their values at a single point in time may not accurately reflect the true situation of patients. Trajectory analysis to identify the different trajectory groups of LAR would be an optional method to solve this problem. Fourth, information on the mechanism and pathogenesis, especially of LDH, is still limited. Firstly, the diagnostic criteria for sepsis and SAKI used in our study do not fully align with those commonly applied in clinical settings, which might limit the practical applicability of our results in a clinical context. Therefore, more well-designed clinical studies, trajectory analysis and basic research are necessary in the future.

## Conclusion

There is a positive linear correlation between the LAR and the risk of developing SAKI in sepsis patients without a history of CKD. Increased LAR was an independent risk factor and had moderate predictive value for SAKI development in the non-CKD sepsis cohort.

### Electronic supplementary material

Below is the link to the electronic supplementary material.

Supplementary Material 1

## Data Availability

ALL data were obtained from the public database MIMIC IV (version 2.1, 10.13026/rrgf-xw32). Structured data for final analysis in the present study are uploaded in supplemental files. More details can be obtained from the corresponding author upon reasonable request.

## Abbreviations

AUC: Area under the curve
BAR: Blood urea nitrogen to albumin ratio
BUN: Blood urea nitrogen
CKD: Chronic kidney disease
ICU: Intensive care units
GCS: Glasgow Coma Scale
IQR: Interquartile range
LAR: Lactate dehydrogenase to albumin ratio
LDH: Lactic dehydrogenase
MAP: Mean arterial pressure
MIMIC IV: The Medical Information Mart for Intensive Care IV
RAR: Red blood cell distribution width-to-albumin ratio
RCS: Restricted cubic spline
ROC: Receiver operating characteristic
SAKI: Sepsis-induced acute kidney injury
SAPS II: Simplified Acute Physiology Score II
Scr: Serum creatinine
SD: Standard deviation
SOFA: Sequential Organ Failure Assessment
SpO2: Oxygen saturation
WBC: Serum white blood cell
