# Supplementary material for: The elevated lactate dehydrogenase to albumin ratio is a risk factor for developing sepsis-associated acute kidney injury: a single-center retrospective study
Source: BMC Nephrol. 2024 Jun 19;25:201. doi: 10.1186/s12882-024-03636-5 (PMC11186243; doi:10.1186/s12882-024-03636-5)
Supplement: Supplementary file 1 — Supplementary Material 1 [file 12882_2024_3636_MOESM1_ESM.docx]

**The elevated lactate dehydrogenase to albumin ratio is a risk factor for developing sepsis-associated acute kidney injury: a single-center retrospective study**

***Supplemental file***

Yipeng Fang ^a,b,c^, Yuan Zhang ^c^, Xin Zhang ^a,b,c^

^a^ Laboratory of Molecular Cardiology, The First Affiliated Hospital of Shantou University Medical College, Shantou, Guangdong Province, People's Republic of China

^b^ Laboratory of Medical Molecular Imaging, The First Affiliated Hospital of Shantou University Medical College, Shantou, Guangdong Province, People's Republic of China

^c^ Shantou University Medical College, Shantou, Guangdong Province, People's Republic of China

Short Title: LAR and developing SAKI

Corresponding Author: Xin Zhang, MD, PhD, Laboratory of Molecular Cardiology, First Affiliated Hospital of Shantou University Medical College 57th Changping Road, Shantou, Guangdong Province, China, 515041; Tel: 0754-88258290; Email: walterzhangx@139.com

**Table S1.** Number of patients with missing data for each variable

| **Variable** | Missing data (n) | Missing data (%) |
| --- | --- | --- |
| Age (years) | 0 | 0 |
| Male (%) | 0 | 0 |
| Ethnicity, white (%) | 0 | 0 |
| Weight (kg) | 33 | <1 |
| Comorbidities |  |  |
| Hypertension (%) | 0 | 0 |
| Coronary heart disease (%) | 0 | 0 |
| Congestive heart failure (%) | 0 | 0 |
| Diabetes mellitus (%) | 0 | 0 |
| Chronic pulmonary disease (%) | 0 | 0 |
| Liver disease (%) | 0 | 0 |
| Chronic kidney disease (%) | 0 | 0 |
| Malignant cancer (%) | 0 | 0 |
| Vital sign |  |  |
| Heart rate (bpm) | 0 | 0 |
| Respiratory rate (bpm) | 0 | 0 |
| MAP (mmHg) | 0 | 0 |
| SpO2 (%) | 0 | 0 |
| Temperature (℃) | 0 | 0 |
| Disease severity score, point |  |  |
| SOFA score | 0 | 0 |
| SAPS II | 0 | 0 |
| GCS | 1 | <1 |
| Laboratory parameters |  |  |
| WBC (k/uL) | 0 | 0 |
| Hemoglobin (g/dL) | 0 | 0 |
| Platelets (k/uL) | 0 | 0 |
| Sodium (mmol/L) | 0 | 0 |
| Potassium (mmol/L) | 0 | 0 |
| Chloride (mmol/L) | 0 | 0 |
| Liver function parameters |  |  |
| Lactic acid (mmol/L) | 557 | 13.6 |
| Bilirubin (mg/dL) | 56 | 1.4 |
| ALT (IU/L) | 49 | 1.2 |
| AST (IU/L) | 43 | 1.1 |
| Creatinine (mg/dL) |  |  |
| Baseline 48hour | 0 | 0 |
| Baseline 7day | 0 | 0 |
| Within 48hour of sepsis diagnosis | 0 | 0 |
| LAR and related parameters |  |  |
| LDH (IU/L) | 0 | 0 |
| Albumin (g/L) | 0 | 0 |
| LAR (IU/g) | 0 | 0 |
| Culture positive |  |  |
| Sputum | 0 | 0 |
| Urine | 0 | 0 |
| Blood | 0 | 0 |
| Vasopressor use (%) | 0 | 0 |

a. Continuous variables are displayed as mean (standard deviation) or median (first quartile–third quartile); categorical variables are displayed as count (percentage); ALT, alanine transaminase; AST, aspartate transaminase; GCS, Glasgow Coma Scale; LAR, lactate dehydrogenase to serum albumin ratio; LDH, lactate dehydrogenase; MAP, mean arterial pressure; SAPS, Simplified Acute Physiology Score; SOFA, Sequential Organ Failure Assessment; SpO2, oxygen saturation; WBC, white blood cell.

**Table S2** The details of variance inflation factor (VIF) in multivariate logistic regression models

|  | Original model | | Adjusted model | |
| --- | --- | --- | --- | --- |
|  | VIF | 1/VIF | VIF | 1/VIF |
| sodium | 99.68 | 0.010032 | 1.92 | 0.519658 |
| potassium | 33.93 | 0.029475 | 2.12 | 0.472568 |
| hemoglobin | 29.08 | 0.034388 | 2.05 | 0.48752 |
| mean blood pressure | 27.34 | 0.036571 | 1.84 | 0.543818 |
| age | 25.99 | 0.038474 | 2.76 | 0.362539 |
| SAPSII score | 19.50 | 0.051279 | 2.77 | 0.360735 |
| GCS score | 12.96 | 0.077136 | 6.60 | 0.151499 |
| sofa score | 6.69 | 0.149455 | 5.58 | 0.179177 |
| platelet | 5.05 | 0.197866 | 4.35 | 0.229827 |
| Alanine transaminase (AST) | 4.84 | 0.206673 | 4.82 | 0.207515 |
| White blood cell | 4.47 | 0.223466 | 4.37 | 0.228772 |
| Aspartate transaminase (ALT) | 3.66 | 0.272903 | 3.65 | 0.274144 |
| LAR value | 2.66 | 0.376011 | 2.62 | 0.38204 |
| lac | 2.64 | 0.379055 | 2.55 | 0.392735 |
| Race | 2.54 | 0.393422 | 2.44 | 0.410469 |
| Male | 2.51 | 0.399178 | 2.40 | 0.41751 |
| Hypertension | 2.10 | 0.477205 | 1.98 | 0.504926 |
| Vasopressor | 2.07 | 0.482245 | 2.02 | 0.494495 |
| Chronic kidney disease (CKD) | 1.98 | 0.505562 | 1.92 | 0.521784 |
| Heart failure (HF) | 1.77 | 0.56477 | 1.75 | 0.571774 |
| Liver disease | 1.72 | 0.582385 | 1.68 | 0.594126 |
| Bilirubin | 1.66 | 0.60102 | 1.65 | 0.60723 |
| Diabetes mellitus (DM) | 1.58 | 0.632374 | 1.57 | 0.635065 |
| Chronic pulmonary disease | 1.41 | 0.711071 | 1.40 | 0.716602 |
| Coronary heart disease (CHD) | 1.39 | 0.717318 | 1.39 | 0.719044 |
| Malignant tumor | 1.31 | 0.762269 | 1.29 | 0.776971 |
| Mean VIF | 11.56 |  | 2.67 |  |
